# Addressing the need for economic modelling when designing and reporting a diagnostic testing accuracy study

**DOI:** 10.3389/fpubh.2025.1729822

**Published:** 2026-01-07

**Authors:** Xuanqian Xie, Jennifer Guo, Jessalyn K. Holodinsky, Rui Fu

**Affiliations:** 1 Acute and Hospital-Based Care, Ontario Health, Toronto, ON, Canada; 2 Institute of Health Policy, Management and Evaluation, University of Toronto, Toronto, ON, Canada; 3 Departments of Emergency Medicine, Community Health Sciences and Clinical Neurosciences, Cumming School of Medicine, University of Calgary, Calgary, AB, Canada; 4 Centre for Health Informatics, Cumming School of Medicine, University of Calgary, Calgary, AB, Canada; 5 O’Brien Institute for Public Health, Cumming School of Medicine, University of Calgary, Calgary, AB, Canada; 6 Alberta Children’s Hospital Research Institute, Cumming School of Medicine, University of Calgary, Calgary, AB, Canada; 7 Hotchkiss Brain Institute, Cumming School of Medicine, University of Calgary, Calgary, AB, Canada; 8 Departments of Community Health Sciences, Surgery and Oncology, University of Calgary, Cumming School of Medicine, Calgary, AB, Canada; 9 The Ohlson Research Initiative, Arnie Charbonneau Cancer Institute, University of Calgary, Calgary, AB, Canada

**Keywords:** diagnostic test, economic evaluations, false negative, false positive, health technology assessment, test accuracy, true prevalence

## Abstract

Model-based economic evaluations rely on results of diagnostic test accuracy (DTA) studies to assess the value-for-money of a new diagnostic or prognostic test. The 30-item Standards for Reporting of Diagnostic Accuracy Studies (STARD) 2015 guidelines are the gold-standard guidelines for DTA studies. In this Perspective paper, we conducted a rapid review of publicly available economic evaluations of a new diagnostic test between 2012 and 2025 to formulate eight elements that we believe could be added to STARD 2015 to help enhance the credibility of DTA-based economic evaluations. Examples from the rapid review and practical recommendations were provided.

## 1 Introduction

Model-based economic evaluations are essential tools in health technology assessment (HTA) to inform funding recommendations for new interventions, including new diagnostic or prognostic tests (termed an index test henceforth) (1). To populate an economic model, data on test performance are derived from diagnostic test accuracy (DTA) studies, which present standard test accuracy measures (e.g., sensitivity and specificity). However, to model the long-term cost and effectiveness of an index test, further information, that is often beyond the scope of a traditional DTA study, may be required. The Standards for Reporting Diagnostic Accuracy (STARD) 2015 statement is one of the gold-standard guidelines for reporting DTA studies (2). However, STARD 2015 has been largely developed for ensuring reporting completeness and transparency in medical research and may not include items that are important for an economic evaluation. In this Perspective article, we proposed additional items for STARD 2015 based on the gaps identified from published economic evaluations. These preliminary findings may have implications on mitigating the gaps between the standard of reporting DTA studies and the conduct of high-quality economic evaluations.

## 2 Study procedures

A rapid review was conducted on the International HTA Database of the International Network of Agencies for Health Technology Assessment^1^ for publicly available reports evaluating an index test using model-based economic evaluation from January 2012 to September 2025. For selected reports, we identified model parameters and/or underlying assumptions that had been recognized to have impacted the final results with limited information from a DTA study. Then, using the 30-item STARD 2015 guideline (2), we delineated whether these missing data had been explicitly mentioned in this guideline; if not, we formulated items that we recommended to be added to STARD 2015 via discussion within the research team. The initial search combining keywords and MeSH headings (such as ‘Diagnostic Screening Programs’) yielded 166 records, of which 25 were relevant after the title/abstract and the full-text screen. We highlighted eight articles (3–10) in this paper.

## 3 Results

This study process yielded eight extended items for STARD 2015 (termed E1-E8, Table 1) including five new items for reporting study results and three new items for reporting the discussion. Below we provide a brief description of each item with rationale and examples obtained from the rapid review.

**Table 1:** Recommended items for extending the STARD 2015 guideline to facilitate the conduct of economic evaluations of a new diagnostic or prognostic test (an index test).

Section and topic	No	Item
Results		
Consequences of false index test results	E1	The health and cost consequences of individuals receiving a false diagnosis from the index test should be clearly described, including when and how the false result can be identified and corrected.
The role of the index test in a diagnostic pathway when multiple tests are involved	E2	The expected or observed role of an index test within an existing diagnostic pathway and the decision rules if multiple tests are involved should be clearly described.
Distribution of other diseases in individuals receiving a negative index test result	E3	For “symptomatic” individuals who receive a negative test result, DTA studies should explore and report the underlying distribution of other diseases in this patient population.
Use of composite reference standard to address an imperfect reference test	E4	It should be clearly stated if an imperfect reference test (e.g., composite reference standard or other methods) is used to evaluate an index test and how the associated limitations are addressed (if applicable).
Health-related quality of life by the index test result and underlying disease status	E5	DTA studies should establish a baseline health-related quality of life (HRQOL) to allow economic evaluations to derive utility values based on the index test result and underlying disease status.
Discussion		
Robust estimation of true disease prevalence	E6	Discussion and/or a sensitivity analysis on the limitations associated with a potentially biased disease prevalence estimate should be provided.
Accuracy of an index test enabled by artificial intelligence (AI)	E7	DTA studies of an AI-enabled index test should report data on model performance over time or comment on the limitations associated with not being able to assess time-dependent accuracy.
Resources used for performing the index test	E8	DTA studies should present data or comment on the resources and costs of performing the index test.

STARD, Standards for Reporting Diagnostic Accuracy; DTA, diagnostic test accuracy; AI, artificial intelligence; HRQOL, health related quality of life.

### 3.1 Consequences of false index test results (E1)

*Description*: The health and cost consequences of individuals receiving a false diagnosis from the index test should be clearly described, including when and how the false result can be identified and corrected.

*Rationale*: While DTA studies present a direct comparison between the index and the reference tests, economic evaluations tend to model the real-world clinical pathway in which generally only one type of test (index or reference) is received by an individual at the time. This means that in order to accurately reflect the disease progression of individuals post-testing, false test results need to be explicitly modelled. We herein recommend DTA studies to report the health consequences of individuals with a false index test result to facilitate economic modelling. Furthermore, the timing of the potential detection and even correction of these false test results should be described. For instance, individuals with a false negative test result may be detected retroactively via the discovery of unexpected disease progression or exacerbations that are deemed to be attributed to a false diagnosis; the incurred losses of health may be quantified in a DTA study. Individuals with a false positive test may be subject to overtreatment, leading to a decrement in the quality of life.

*Example*: In a systematic review of economic evaluations on hand-held electrochemical devices for monitoring fractions of exhaled nitric oxide for the diagnosis of asthma (3), the omission of health consequences associated with an incorrect diagnosis was identified to be a major limitation of the published studies. A primary economic evaluation was then performed to overcome this limitation; however, given the paucity of data in the DTA literature, expert opinions were used to determine key model parameters including the number of years required to detect and correct a false diagnosis result, rendering the final modelling findings to be uncertain.

### 3.2 The role of the index test in a diagnostic pathway when multiple tests are involved (E2)

*Description*: The expected or observed role of an index test within an existing diagnostic pathway and the decision rules if multiple tests are involved should be clearly described.

*Rationale*: To arrive at a final diagnosis, individuals are sometimes exposed to more than one type of test (11). When an index test is part of a diagnostic pathway with other tests, the DTA study should clarify or propose the role of the index test as the following (12): a replacement (i.e., replacing one or more existing tests due to an increase in diagnostic accuracy), a triage (i.e., preceding the conduct of existing tests for risk stratification so that some individuals may not require the subsequent tests), or an add-on (i.e., to be performed as an additional test in the existing diagnostic pathway). In the presence of multiple tests (11), an ‘OR’ rule may be applicable on some occasions where a positive diagnostic result is reached given any positive individual test result. In other instances, a positive diagnostic result may only be reached if all individual tests are positive (i.e., the ‘AND’ rule). When the results of multiple tests are strongly correlated due to an underlying dependence structure (13), the DTA study may want to report such pairwise correlations and discuss the possibility of omitting one of these tests to streamline the diagnostic pathway.

*Example*: In 2024, an economic model (4) was published comparing the budget impact of adopting four diagnostic pathways of latent tuberculosis infection involving an interferon-gamma release assay (IGRA) and/or the standard tuberculin skin test (TST), including: i) TST alone; ii) IGRA alone; iii) IGRA following a positive TST result where those tested positive in both tests were deemed to have the disease (i.e., applying the ‘AND’ rule); and iv) TST following a negative IGRA result where individuals receiving a positive result in any test were diagnosed (i.e., applying the ‘OR’ rule). Compared with single-test strategies (i or ii), the ‘AND rule in strategy iii was associated with a lower probability of being treated and lower per-person costs, while the ‘OR’ rule in strategy iv resulted in a higher probability of being treated and higher per-person costs.

### 3.3 Distribution of other diseases in individuals receiving a negative index test result (E3)

*Description*: For “symptomatic” individuals who receive a negative index test result, DTA studies should explore and report the underlying distribution of other diseases in this patient population.

*Rationale*: Many symptoms such as cough and headache are associated with more than one possible underlying diseases. As such, an individual in receipt of a correct negative result from the index test (i.e., true negative) may have other undiagnosed health conditions with both cost and quality-of-life implications. In an economic model, these individuals are typically assumed to enter the “healthy (disease-free)” state following a true negative index test result. Until data are made available from DTA studies to illustrate the underlying distribution of diseases in this heterogenous patient population, a comprehensive modelling of the clinical pathway is not feasible.

*Example*: Asthma, chronic obstructive pulmonary disease (COPD) and congestive heart failure (CHF) are distinct diseases that share many symptoms including the shortness of breath, while the latter two diseases are associated with considerably higher health and cost burdens. In a 2017 economic evaluation on diagnostic tests for asthma (5), the possibility of individuals tested negative for asthma but otherwise had COPD or CHF was entertained in the economic model. However, due to the paucity of objective data on the distribution of COPD/CHF in this patient population, only expert opinions were used to populate the model.

### 3.4 Use of composite reference standard to address an imperfect reference test (E4)

*Description:* It should be clearly stated if an imperfect reference test (e.g., composite reference standard or other methods) is used to evaluate an index test and how the associated limitations are addressed (if applicable).

*Rationale:* An imperfect reference test is used to evaluate an index test when a perfect test (i.e., the gold-standard test for determining disease status) is unavailable (14). Due to the inherent limitations of an imperfect reference test, a biased estimation on the accuracy of the index test may arise. For example, despite an index test producing the correct diagnosis for an individual, a DTA study relying on an imperfect reference test may render that diagnosis to be incorrect/false, and thereby introduces errors in the subsequent economic evaluation. To address this limitation, one approach is to assess the index test against a composite reference standard, which classifies individuals into disease positive or negative groups using multiple imperfect tests, though this method has its own limitations related to the uncertainty of the true disease prevalence (15). More sophisticated methods (e.g., latent-class models) are emerging to tackle this issue (14, 15).

*Example:* Diagnosis of community-acquired pneumonia caused by *Streptococcus pneumoniae* in adults is often based on a composite reference standard using culture methods (blood culture, sputum, and cultures of other respiratory samples) (10). The BinaxNOW *S. pneumoniae* (BinaxNOW-SP, the index test) is a urine-based test that can potentially achieve a faster and better diagnosis. In a 2013 review of 27 DTA studies (10), a Bayesian latent-class meta-analysis suggested that the sensitivity of BinaxNOW-SP was higher than the composite reference standard, while both achieved high specificities. An economic evaluation was further conducted using the meta-analyzed test accuracy of the BinaxNOW-SP to improve the precision of the modelling results (6).

### 3.5 Health-related quality of life by the index test result and underlying disease status (E5)

*Description:* DTA studies may want to establish a baseline health-related quality of life (HRQOL) to allow economic evaluations to derive utility values based on the index test result and underlying disease status.

*Rationale:* Due to the paucity of health utility (HRQOL) data, health economists are confronted with the challenge of assigning a utility value to the population based on the index test result and the underlying disease status (i.e., true/false positive and negative groups; a total of four groups). The common approach is to start with a “baseline” HRQOL value (typically for the true-negative group) using the utility of the general disease-free population (16, 17), and then be able to adjust that utility for the other three groups by incorporating an appropriate decrement. However, this approach has several pitfalls including not being able to account for individuals who tested negative but are otherwise living with other health conditions (see section 3.3) and incorrectly classifying individuals (and thereby assigning the ‘wrong’ utility) due to the use of an imperfect reference test (see section 3.4). Also, the symptom burden for the true-positive and false-negative groups may be different, although both are categorized as having the disease in an economic model and likely will share the same utility value to capture HRQOL. We thereby recommend DTA studies to provide data that can define the baseline HRQOL of the patient population and provide suggestions on the best way to estimate the HRQOL based on the index test result and the underlying disease status.

*Example:* In the asthma example that we previously presented in section 3.1 (3), individuals receiving a negative index test for asthma (including true and false negatives) were assumed to have the same health utility value as the healthy population, while those who received a false positive result were assumed to experience the same disutility as those with true underlying asthma condition (i.e., true positives). These assumptions may lead to uncertainties in the final modelling results.

### 3.6 Robust estimation of true disease prevalence (E6)

*Description:* Discussion and/or a sensitivity analysis on the limitations associated with a potentially biased disease prevalence estimate should be provided.

*Rationale:* Disease prevalence is key to determine the cost-effectiveness of an index test compared to the standard of care; however, in many cases the true prevalence of disease cannot be reliably estimated. Specifically, individuals with a negative test result are often not investigated further (e.g., those tested negative in cancer screening), and consequently, the true disease status of these individuals (whether they are a true or false positive in the first place) remains unknown (18), leading to an underestimated disease prevalence. Furthermore, in the absence of a perfect reference standard, the calculation using the accuracy of an index test ([TP + FN]/[TP + FN + FP + TN]) may not reflect the true prevalence of the disease (15). Advanced methods including latent-class modeling (15) are emerging to reduce bias in estimating disease prevalence and may be adopted in the reporting of DTA studies.

*Example:* An economic evaluation was conducted on noninvasive prenatal testing for trisomy 21 (Down syndrome) at 12-week gestation in 2019 (7). To estimate the true prevalence of trisomy 21 at 12 weeks, the report used the prevalence of trisomy 21 in live birth and the rate of spontaneous pregnancy loss from 12 weeks to term due to the chromosomal anomaly to arrive at an estimation. This estimated prevalence was ultimately used in the economic evaluation.

### 3.7 Accuracy of an index test enabled by artificial intelligence (AI; E7)

*Description*: DTA studies of an AI-enabled index test should report data on model performance over time or comment on the limitations associated with not being able to assess time-dependent accuracy.

*Rationale*: While most economic evaluations assume the performance of the index test to be constant over the modelled time horizon, there is an emerging class of diagnostic systems powered by AI with improving or degrading performance over time (19, 20). Until adequate information is made available on how often these algorithms are expected to be updated, how these updates will occur, and the time-dependent change of the underlying algorithm (i.e., learning curves), a comprehensive economic evaluation is not feasible (21).

*Example*: A 2022 evaluation found an AI-assisted computed tomography (CT) scan protocol for lung cancer screening in high-risk adults to be cost-effective over radiologist-interpreted CT scans for the healthcare system over a 20-year time horizon (8). The sensitivity and specificity of the two protocols were obtained from a single DTA study (22) and assumed to remain constant over time. Interestingly, this DTA study also found the underlying deep learning algorithms to be superior to radiologists only during the initial screening, but became on-par when radiologists had access to previous examinations. These results were not incorporated into the economic evaluation and thus may imply the cost-effectiveness results to be overly optimistic favoring the AI-assisted CT scan protocol in a longitudinal screening setting.

### 3.8 Resources used for performing the index test (E8)

*Description*: DTA studies should present data or comment on the resources and costs of performing the index test.

*Rationale*: In an economic evaluation, the listing prices of products and services (e.g., technical support and maintenance agreement) obtained from the manufacturers are typically used to estimate the cost of an index test. This method has likely resulted in an underestimation as other cost components including infrastructure depreciation or new purchases, professional fees paid to technicians and other personnel, fees of obtaining the license or specialized software, and other operation costs, are not considered. For example, hospitals that need to establish a new lab (and recruiting personnel) to perform an index test may incur substantial upfront expenditures. We recommend investigators to present this information in a DTA study.

*Example*: In an economic evaluation of a DNA methylation–based classifier test for central nervous system tumors (9), the capital costs were not included in the reference-case analysis. The inclusion of capital costs (e.g., purchase and equip the entire platform and obtain the license to use the technology) would substantially inflate the cost of testing, and thereby resulting in a less favorable cost-effectiveness profile for the index test. This index test is also powered by a random forest (a type of machine learning algorithm) classification model, which introduces further complexity on how to properly evaluate its cost-effectiveness against the usual care, given the lack of primary DTA data and analytical guidance (see section 3.7).

## 4 Discussion

DTA studies often provide the foundational data to inform an economic evaluation of an index test. Through a structured process, we provided recommendations on five additional items that DTA investigators may want to assess as secondary outcomes (items E1 to E5) and three additional items to be elaborated in the discussion, if primary data collection/analysis is not feasible (items E6 to E8). Note that some of these recommended items are not entirely independent from the original items in STARD 2015 but we provided more specific and actionable instructions with the goal of improving the appropriate use of DTA data in economic evaluations. For example, the STARD 2015 guidelines recommend the reporting of “the intended use and clinical role of the index test” in the background and discussion sections of a DTA study (2). Built upon this, our item E2 further specified that DTA studies may want to explicitly present the decision rules involving the index test in a diagnostic pathway and report the conditional dependence between tests. Similarly, the STARD 2015 has called for a description on the reference standard “in sufficient details to allow replication” (2); in our item E4, we further clarified that in the case of using an imperfect reference standard, DTA researchers should explicitly discuss the associated limitations and consider exploring more sophisticated methods to minimize impact. In this paper, we did not elaborate on the implementation of our proposed items especially for E6–E8 that we considered to be more difficult to examine. For example, in item E7 we recommend DTA studies to discuss the implications of time-dependent performance of an AI-enabled index test even if these longitudinal data are difficult to obtain due to barriers in implementing AI algorithms, even for pilot testing (23). This recommendation also aligns with regulations such as those from the US Food and Drug Administration who only evaluates AI tools with a locked algorithm and requires new applications whenever the algorithm has been updated (24). Overall, we recommend health economists and health decision scientists to actively participate in the creation of DTA reporting guidelines to ensure the inclusion of essential data elements for an economic evaluation.

## Data Availability

The original contributions presented in the study are included in the article/supplementary material, further inquiries can be directed to the corresponding author.

## Footnotes

1
https://database.inahta.org/
